# The role of psychotropic medication, alcohol, illicit drugs, and suicidal intention in fatal motor vehicle accidents involving drivers with psychotic disorders

**DOI:** 10.1093/eurpub/ckaf263

**Published:** 2026-02-16

**Authors:** Jussi Koskinen, Helinä Hakko, Pirkko Riipinen, Niina Sihvola, Anu-Helmi Halt

**Affiliations:** Research Unit of Clinical Medicine, Psychiatry, University of Oulu, Oulu, Finland; Research Unit of Population Health, University of Oulu, Oulu, Finland; Department of Psychiatry, Oulu University Hospital, Oulu, Finland; Research Unit of Clinical Medicine, Psychiatry, University of Oulu, Oulu, Finland; Department of Psychiatry, Oulu University Hospital, Oulu, Finland; Finnish Crash Data Institute (OTI), Helsinki, Finland; Research Unit of Clinical Medicine, Psychiatry, University of Oulu, Oulu, Finland; Department of Psychiatry, Oulu University Hospital, Oulu, Finland; Medical Research Center, Oulu University Hospital and University of Oulu, Oulu, Finland

## Abstract

Antipsychotics, antidepressants, and benzodiazepines are commonly used among patients with psychotic disorders. The use of psychotropic medication, alcohol, and illicit drugs may have a major role in the fatal motor vehicle accidents (FMVA) involving drivers with psychotic disorders. This study on drivers involved in FMVAs in Finland investigates the post-mortem toxicology findings regarding the presence of psychotropic medication, alcohol and illicit drugs among 94 drivers with psychotic disorders and their 188 matched controls without psychiatric disorders. Drivers’ suicidal intention, death category, and role in the accident were also investigated. Psychotropic medication was present in 53.2% of drivers with psychotic disorders and in 9.6% of controls at the time of their FMVA. Among the drivers with psychotic disorders, the presence of antipsychotics was detected in 30% of them, and their suicidal intentions behind FMVA were significantly associated with the presence of antipsychotics (OR 2.7) and mood stabilizers (OR 6.0). Our results suggest that, in some cases, traffic suicides among drivers with psychotic disorders may originate from long-term suicidal thoughts and occur despite antipsychotic medication. A thorough fitness-to-drive assessment should be conducted for patients with psychotic disorders, irrespective of their antipsychotic medication status.

## Introduction

Schizophrenia is often associated with poor driving ability [1, 2]. The use of antipsychotics, antidepressants, and benzodiazepines is common in psychotic disorders [3]. Psychotropic medication is shown to associate with the likelihood of being involved in a traffic accident [4]. In addition, the driving ability of patients with schizophrenia may be impaired regardless of antipsychotic medication [5]. Keski-Filppula *et al.* [6] reported that 43% of 94 drivers with a psychotic disorder killed in fatal motor vehicle accidents (FMVAs) in Finland between 1990 and 2011 were taking medication with the potential to affect driving ability at the time of the FMVA.

A systematic review of longitudinal studies on suicidality in first-episode psychosis (FEP) emphasized the importance of early detection and continuing assessment of suicidal risk [7]. Among 14 907 study subjects with follow-up varying periods from 1 to 42 years, 27% had reported suicidal ideation, 22% had attempted suicide and 1%–4% died by suicide. In a Finnish study of 264 individuals with a lifetime history of any psychotic disorder, excluding those caused by a general medical condition, almost 35% had attempted suicide [8]. Furthermore, a study of fatal motor vehicle accidents (FMVAs) in Finland between the years 1990 and 2011 found a significant association between being a driver with a psychiatric disorder and committing suicide while driving (OR 3.4 for males and 3.8 for females) [9].

This study extends earlier research on the 94 FMVA drivers with psychotic disorders by investigating the findings of their post-mortem toxicology analyses. The control group is comprised of within sample matched FMVA drivers without any psychiatric disorders.

## Methods

### Data sources

The present study utilizes the nationwide registry-based database from the Finnish Crash Data Institute (OTI), with a linkage to the Care Register for Health Care (CRHC) and the National Causes of Death Register.

The OTI database and additional paper documents placed in accident folders for each fatal motor vehicle accident (FMVA) contain comprehensive information on drivers who were killed in these accidents in Finland, and whose accidents were investigated by the Finnish Road Accident Investigation Teams (RAITs). Detailed information on the work of the RAITs is outlined on the official website of the OTI [10].

Data on driver’s inpatient episodes (from 1969 onwards) and specialized outpatient visits (from 1998 onwards) was extracted from the CRHC, administered by the Finnish Institute for Health and Welfare [11]. The CRHC records diagnoses using the International Classification of Diseases (ICD), with three different versions of ICD used in Finland since 1969 (ICD-8 from 1969 to 1986, ICD-9 from 1987 to 1995, and ICD-10 from 1996 onwards).

The official causes-of-death data were obtained from the Causes of Death Register, hosted by Statistics Finland [12]. The cause-of-death diagnoses are also recorded using the ICD classification. Police investigations to determine the cause of death are conducted if the death was caused by a crime, accident, or suicide or if the death occurred unexpectedly. A forensic autopsy is usually performed when establishing the cause-of-death requires a police investigation. The forensic cause-of-death investigation may also include additional analyses, including post-mortem toxicology analyses.

### Medico-legal findings

Information on the presence of medicine, alcohol, or illicit drugs at the time of FMVA was based on the post-mortem toxicology documents and other information recorded in the OTI database and in the additional paper documents (statements on the cause of death and paper documents filled out by RAIT members).

Toxicology examination routines may vary worldwide [13], and methods used in Finland have been described, for example, in an article written by Launiainen and Ojanperä [14]. Based on clinical practice, it was assumed that if illicit drug analyses were performed, autopsy samples were also tested for medication, even if the results of medication analyses were not reported in any of the available documents. Where information on medication analyses was available, but any record of illicit drug was absent, it was not assumed that illicit drugs analyses had been performed. After accounting for these assumptions, from the total study sample of 282 drivers, information on post-mortem toxicology analyses regarding drivers’ medication in autopsy samples at the time of FMVA was not available for 61 drivers (14 in cases and 47 in controls). Information on alcohol was missing for three drivers and for illicit drugs for 131 drivers.

Psychotropic medication is comprised of antipsychotics, antidepressants, mood stabilizers, anxiolytics, hypnotics, and sedatives, according to the Anatomical Therapeutic Chemical Classification [15]. One benzodiazepine drug, clonazepam, was excluded from the psychotropic medication subgroup due to its ATC-classification as an antiepileptic medicine. Clonazepam without other benzodiazepines was found only in one autopsy sample. Further, lithium was excluded from the antipsychotics subgroup due to its primary use as a mood stabilizer. The subgroup of mood stabilizers consisted of lithium from antipsychotics, and carbamazepine/hydroxy-carbamazepine, lamotrigine, oxcarbazepine, and valproic acid from antiepileptics.

Alcohol levels were measured using blood and/or other samples. One blood sample with a positive post-mortem but with a negative antemortem result was defined as negative due to the probable post-mortem ethanol production. Otherwise, drivers were categorized based on the highest alcohol concentration recorded in any autopsy sample. Driving Under the Influence of Alcohol (DUIA) at the time of FMVA was analysed using the following categories of alcohol concentration: (i) under 0.5‰, (ii) DUIA, 0.5‰–1.19‰ (i.e. limit for drunken driving in Finland) and (iii) aggravated DUIA, 1.2‰ or more (i.e. limit for aggravated drunken driving in Finland). In logistic regression analyses, drivers were categorized into two groups: (i) no DUIA (under 0.5‰;) and (ii) DUIA, 0.5‰ or more.

Illicit drugs is comprised of cannabis, amphetamine, and methamphetamine. The presence of cannabis metabolites tetrahydrocannabinol and tetrahydrocannabinolic acid, amphetamine, or methamphetamine have been detected in blood and/or urine samples. Methamphetamine was detected in only one blood sample together with amphetamine. Opioids were analysed separately, because the opioids found in our data may also have been used as analgesics.

### Study population

Based on the OTI database, a total of 4281 Finnish drivers (aged between 16 and 93 years) had died in motor vehicle accidents in Finland between the years 1990 and 2011. Of these, based on CRHC, 94 drivers were diagnosed as having a psychotic disorder (ICD-10-codes: F20, F22–F25 and F28–F29; ICD-8/9 diagnoses converted to ICD-10 codes) during the 5 years prior to their FMVA. Drivers with two or less specialized outpatient visits due to a psychotic disorder and those diagnosed with only substance use related psychosis were excluded from our final study sample (cases). Of the 94 drivers with a psychotic disorder deemed eligible, 28.7% were diagnosed with schizophrenia (F20), 41.5% with other specified psychoses (F22–F25), and 29.8% with unspecified psychoses (F28–F29) [6].

Using the post-mortem findings on antipsychotics, 94 drivers with a psychotic disorder were categorized into (i) Drivers with a psychotic disorder taking antipsychotic medication (PDA, *n* = 28), and (ii) Drivers with a psychotic disorder without antipsychotic medication (PDNA, *n* = 66). The control group (*n* = 188) consists of within sample matched FMVA drivers without any history of inpatient episodes or specialized outpatient visits in the CRHC due to any psychiatric disorder during the 10 years prior to their FMVA. Two controls for each case of deceased drivers with a psychotic disorder were selected and matched by age (±3 years), sex and the year of the FMVA (±3 years).

### Driving and accident-related variables

Drivers’ role in fatal accidents, based on the evaluation of the RAIT police member, was analysed in three categories: (i) at-fault driver, (ii) other participant, and (iii) single vehicle accident. The at-fault driver is defined by the police authorities as the driver whose actions contributed most to the occurrence of a fatal accident involving two or more motor vehicles. Correspondingly, the contribution of other participants is considered secondary compared to that of the at-fault driver. Single vehicle accidents refer to collisions involving only one motor vehicle, such as run-off-road incidents or collisions with animals.

Based on driving licence status in the police registers (OTI data), drivers were categorized into two groups: (i) valid driving licence and (ii) no valid driving licence. The latter group includes drivers who have never held a driving licence, those whose driving licence had expired, those who were currently banned from driving, those who had a valid driving licence for another vehicle class, and cases where driving licence status was unknown.

Five different official death categories, collected from the Cause of Death Register, were used for our data (accident, disease, no death certificate, suicide, undetermined intent). Information on drivers’ suicidal intentions was collected from OTI data. Based on the police investigation, a RAIT police member assesses the actions taken by a motor vehicle driver to avoid a fatal accident. The present study focused on cases in which the available evidence indicated that suicidal intent was the reason the driver did not attempt to avoid the accident. We also investigated whether the participant was assessed by the RAIT police member as having experienced fatigue (was the driver tired at time of FMVA: yes, no/not known) or strain (physical, mental or other forms of stress) at the time of fatal accident.

### Statistical analysis

The statistical significance of group differences was assessed using Pearson Chi-Square or Fisher-Freeman-Halton Exact test, where appropriate. Logistic regression was used to examine the association of the presence of psychotropic medication, alcohol, illicit drugs, or opioids at the time of fatal accident with cause-of-death category and suicidal intention behind FMVA. Each subgroup of psychotropic medication in our study, alcohol and illicit drugs were entered, separately, into logistic regression models as exposure variables (substance found in post-mortem toxicology analyses = 1, substance not found = 0). Outcome variables included death categories (suicide = 1 vs. other death categories = 0; accident = 1 vs. other death categories = 0) and suicidal intention behind FMVA (yes, no). Odds ratios (ORs) with 95% confidence intervals (95% CIs) are reported for suicides and accidents as the official cause of death, and for suicidal intentions behind FMVA in drivers with the presence of medication, alcohol, or illicit drug at the time of their fatal accident versus drivers without medication, alcohol or illicit drugs. ORs were age- and sex-adjusted for the 94 drivers with a psychotic disorder and unadjusted for all 282 drivers of the study population. IBM SPSS Statistics 28 software was used for statistical analyses.

**Table 1.** The presence of psychotropic medication, alcohol, illicit drugs, and opioids at the time of fatal motor vehicle accident (FMVA) in the total study sample of 282 drivers, and by study groups, *n* (%).

Findings of the post-mortem toxicology analyses^a^	Total	Study groups			Statistical difference between study groups, *P* value
		Psychotic disorder, antipsychotic medication (PDA)	Psychotic disorder, no antipsychotic medication (PDNA)	Controls	
	(*n = *282)	(*n = *28)	(*n = *66)	(*n = *188)	
Psychotropic medication, any, *n* (%)	68 (24.1)	28 (100)	22 (33.3)	18 (9.6)	<.001
Antipsychotics	28 (9.9)	28 (100)	0 (0)	0 (0)	<.001
Antidepressants	22 (7.8)	9 (32.1)	9 (13.6)	4 (2.1)	<.001
Anxiolytics, hypnotics and sedatives	38 (13.5)	7 (25.0)	17 (25.8)	14 (7.4)	<.001
Benzodiazepines	34 (12.1)	5 (17.9)	16 (24.2)	13 (6.9)	<.001
Other	4 (1.4)	2 (7.1)	1 (1.5)	1 (0.5)	.032
Mood stabilizers	11 (3.9)	6 (21.4)	3 (4.5)	2 (1.1)	<.001
Driving under the influence of alcohol (DUIA) (‰)^b^, *n* (%)					.004
No DUIA (0‰–0.5‰)	202 (71.6)	27 (96.4)	52 (78.8)	123 (65.4)	
DUIA (0.5‰–1.19‰)	7 (2.5)	0 (0)	1 (1.5)	6 (3.2)	
Aggravated DUIA (1.2‰ or more)	73 (25.9)	1 (3.6)	13 (19.7)	59 (31.4)	
Illicit drugs, *n* (%)	9 (3.2)	2 (7.1)	2 (3.0)	5 (2.7)	.398
Amphetamine/Methampheatmine	5 (1.8)	1 (3.6)	1 (1.5)	3 (1.6)	.605
Cannabis	7 (2.5)	2 (7.1)	1 (1.5)	4 (2.1)	.198
Opioids, *n* (%)	5 (1.8)	0 (0)	1 (1.5)	4 (2.1)	1.000

a Based on the post-mortem toxicology documents or other investigation documents of FMVA accident folders (The Finnish Crash Data institute, https://www.lvk.fi/en/the-finnish-crash-data-institute-oti/oti/).

b Alcohol concentration in post-mortem samples (‰).

**Table 2.** Role in the accident, driving licence status, official cause-of-death category, suicidal intentions behind FMVA and fatigue or strain at time of accident among the total study sample of 282 FMVA, and by study groups, *n* (%).

	Total	Study groups			Statistical difference between study groups, *P* value
		Psychotic disorder, antipsychotic medication (PDA)	Psychotic disorder, no antipsychotic medication (PDNA)	Control group	
*n* (row-% of total *n = *282)	282 (100)	28 (9.9)	66 (23.4)	188 (66.7)	
Role in the accident, *n* (%)					<.001
At-fault driver	144 (51.1)	23 (82.1)	46 (69.7)	75 (39.9)	
Other participant	43 (15.2)	1 (3.6)	3 (4.5)	39 (20.7)	
Single-vehicle accident	95 (33.7)	4 (14.3)	17 (25.8)	74 (39.4)	
Valid driving licence, *n* (%)	259 (91.8)	28 (100)	52 (78.8)	179 (95.2)	<.001
Official cause-of-death category, *n* (%)					<.001
Suicide	35 (12.4)	10 (35.7)	16 (24.2)	9 (4.8)	
Accident	211 (74.8)	12 (42.9)	37 (56.1)	162 (86.2)	
Disease	9 (3.2)	0 (0)	2 (3.0)	7 (3.7)	
Undetermined intent	26 (9.2)	6 (21.4)	11 (16.7)	9 (4.8)	
No death certificate	1 (0.4)	0 (0)	0 (0)	1 (0.5)	
Suicidal intention behind FMVA, defined by the road-accident investigation team, *n* (%)^a^	43 (15.2)	13 (46.4)	18 (27.3)	12 (6.4)	<.001
Fatigue, *n* (%)	50 (17.7)	3 (10.7)	11 (16.7)	36 (19.1)	0.529
Strain, *n* (%)	53 (18.8)	5 (17.9)	12 (18.2)	36 (19.1)	0.976

a Based on the interviews and other background material of OTI data (The Finnish Crash Data institute, https://www.lvk.fi/en/the-finnish-crash-data-institute-oti/oti/).

**Table 3.** The association of the presence of psychotropic medications, alcohol or illicit drugs at the time of fatal motor vehicle accident (FMVA) with likelihood of suicide or accident as official cause-of-death category and suicidal intentions behind FMVA, among 94 drivers with psychotic disorders.

Finding of the post-mortem toxicology analyses^a^	Official cause-of-death category				Suicidal intentions behind FMVA, defined by the road-accident investigation team, (yes vs. no)^b^	
	Suicide vs. other death categories		Accident vs. other death categories			
	OR (95% CI) ^c^	*P*	OR (95% CI)^c^	*P*	OR (95% CI)^c^	*P*
Psychotropic medication						
Antipsychotics	1.88 (0.70–5.03)	.212	0.52 (0.20–1.34)	.176	2.65 (1.00–7.01)	.050
Antidepressants	1.81 (0.56–5.82)	.322	0.66 (0.22–2.02)	.468	1.90 (0.60–6.01)	.276
Anxiolytics, hypnotics and sedatives	1.11 (0.36–3.41)	.860	1.33 (0.49–3.62)	.583	1.48 (0.51–4.34)	.472
Mood stabilizers	3.40 (0.61–19.05)	.165	0.25 (0.05–1.23)	.088	5.96 (1.05–33.72)	.044
Driving under the influence of alcohol (DUIA), yes	ne^d^		28.52 (3.36–242.16)	.002	0.20 (0.04–0.99)	.048
Illicit drugs, yes	ne		4.93 (0.47–52.23)	.185	ne	
Opioids, yes	ne		ne		ne	

a Based on the post-mortem toxicology documents or other investigation documents of FMVA accident folders (The Finnish Crash Data institute, https://www.lvk.fi/en/the-finnish-crash-data-institute-oti/oti/).

b Based on the interviews and other background material of OTI data (The Finnish Crash Data institute, https://www.lvk.fi/en/the-finnish-crash-data-institute-oti/oti/).

c Age- and sex-adjusted odds ratio (OR) with 95% confidence interval (CI).

d ne (not estimable) means the number of cases in these categories was too small for statistical analysis.

## Results

The post-mortem findings of FMVA drivers with psychotic disorders, compared to the controls, were related to any psychotropic medication (53.2% vs. 9.6%), antipsychotics (29.8% vs. none), antidepressants (19.1% vs. 2.1%), anxiolytics, hypnotics and sedatives (25.5% vs. 7.4%), and mood stabilizers (9.6% vs 1.1%). Table 1 shows the presence of psychotropic medication, alcohol, illicit drugs and opioids at the time of FMVA, by PDA and PDNA study groups of drivers and controls. The presence of anxiolytics, hypnotics, and sedatives was about 25% in both the PDA and PDNA groups. Further, 19.7% of drivers in the PDNA group and 31.4% of the controls had driven under the influence of alcohol, with alcohol concentration being 1.2‰ or higher (aggravated DUIA), while nearly all drivers in the PDA group belonged to the group of no-DUIA. Among all 282 FMVA drivers, illicit drugs were recorded for 3.2% and opioids for 1.8%. Details of the medications identified in post-mortem toxicology analyses are presented in Supplementary Table S1.

Table 2 presents the driver’s role in the accident, driving licence status, official cause-of-death category, and suicidal intentions behind FMVA, by study groups. The proportion of at-fault drivers was notably higher in the PDA (82.1%) and PDNA (69.7%) groups than in the controls (39.9%). Driving licences were valid for all drivers in the PDA group, 95.2% of the controls, and 78.8% of the drivers in the PDNA group. Suicide as official cause-of-death category was higher in the PDA (35.7%) and PDNA groups (24.2%) compared to the controls (4.8%). Driver’s suicidal intentions behind FMVA were higher in the drivers of the PDA (46.4%) and PDNA (27.3%) groups than in the controls (6.4%). The proportion of drivers assessed as having been tired or experiencing strain at the time of FMVA did not differ significantly between the study groups.

The main findings are presented in Table 3, showing associations of the presence of psychotropic medication, alcohol, illicit drugs and opioids at the time of FMVA with the likelihood of having suicide or accident as the official cause-of-death category, and with suicidal intention behind FMVA. Among the sample of 94 FMVA drivers with psychotic disorders, no significant association was observed between medication, alcohol, and illicit drugs and suicide (compared to other cause-of-death categories). Instead, accident as cause-of-death category was significantly associated with driving under the influence of alcohol (DUIA) (OR 28.5, *P = .*002). Further, suicidal intention behind FMVA, compared to those without it, was more likely among drivers with the presence of antipsychotics (OR 2.7, *P = .*050) and mood stabilizers (OR 6.0, *P = .*044), whereas driving under the influence of alcohol (DUIA) was less likely (OR, 0.2–0.048). Corresponding analysis for the total sample of 282 drivers is presented in Supplementary Table S2.

## Discussion

The key finding of this study was that, among drivers with psychotic disorders, suicidal intentions behind FMVA were significantly associated with the presence of antipsychotics and mood stabilizers at the time of the FMVA. Reasons for road traffic suicides may often remain unclear [16]. A recent study reported that the time from suicidal ideation to an actual suicide attempt is typically short—often only a matter of hours [17]. In the current study, however, the vast majority of deceased drivers with psychotic disorders, who had used antipsychotics, were not under the influence of alcohol at the time of their FMVA. This suggests that violent traffic suicides involving drivers with psychotic disorders are not necessarily impulsive and may, in some cases, result from long-term suicidal ideation and occur despite treatment with appropriate medication. Conversely, FMVAs involving drivers with psychotic disorders who were not taking antipsychotic medication were more frequently classified as accidents and one-fifth of the cases involved aggravated drunk drivers. A meta-analysis [18] found that among psychiatric study samples, the prevalence of current passive suicidal ideation (i.e. desire for death or thought of death) was 34%, while the lifetime prevalence was 47%. Researchers have also noted that the clinical importance of passive ideation in suicide risk assessment has remained unclear.

Generally, antipsychotic medication was only found to be present in one-third of drivers with psychotic disorders. This may indicate problems of medical nonadherence in patients with psychotic disorders [19, 20]. Of the drivers with psychotic disorders, all of those taking antipsychotics and 80% of those without antipsychotics had a valid driving licence, according to the police-register. A Fitness-to-drive assessment is a medical evaluation conducted by health care professionals and is one of the methods available for the prevention of severe traffic accidents. Unfortunately, short-term driving bans (under 6 months) imposed by a physician are only recorded in the patient’s medical records and not as a variable in the OTI data and thus this information was not available in our study. An earlier study of 94 FMVA drivers with psychotic disorders [6] reported that almost half of them were discharged from psychiatric care during the 3 months prior to their FMVA. Further, among these 94 drivers, 74% of those with comorbid depression had died in an FMVA within 6 months following their last psychiatric discharge [21].

The number of all fatal traffic accidents in Finland has remained relatively low during recent years. During our study period (years 1990–2011), 4281 Finnish drivers (aged between 16 and 93 years) died in FMVAs. According to The Fit-to-Drive Report of Finnish Crash Data Institute, during the years 2014–18 a total of 907 fatal accidents was investigated by road accident investigation teams [22]. However, the role of psychiatric disorders and substance use in these accidents is significant; 39% of those 907 fatal accidents involved a medical condition as a background risk factor and the most common medical conditions were substance dependence (*n* = 217) and mental illness (*n* = 162).

Of all fatal 4281 FMVAs during our study period, only under 7% were officially classified as suicides [9]. However, the role of psychotic disorders in suicidal traffic deaths is notable, as almost 30% of FMVAs involving drivers with psychotic disorders of our data were classified as suicides. It is also possible that traffic deaths occurring in consequence of suicidal intention in our study are underestimated due to difficulty in reliably identifying all such cases [23, 24]. Further research on traffic suicides, with a focus on certain driver groups (e.g. young drivers) would be valuable. In addition, focusing also on those traffic suicidal attempts which have not caused a death of a driver, would offer more information and data to work with and potentially reveal more factors behind this phenomenon.

### Strengths and limitations

Comprehensive accident data of OTI with a linkage to national registers is a major strength of our study. Another strength of our study is that, based on post-mortem toxicology examinations, we were able to evaluate the presence of psychotropic medication, drugs, and alcohol at the time of fatal accident. One limitation is that the post-mortem toxicology analyses were not uniformly available for each FMVA driver in our study and, in addition, the possibility of false negatives should also be noted [25]. The possibility of medication withdrawal as a potential contributor to fatal motor vehicle accidents needs to be considered; the use of antipsychotics and certain other psychotropics pose a risk of post-withdrawal disorders and potential high severity of symptoms [26]. In our data it was, unfortunately, not possible to evaluate medical withdrawals, dose adjustments or recently started new medication which may affect the driving ability.

## Conclusion

Results of this study indicate that, among drivers with psychotic disorders, antipsychotic medication does not eliminate the need to carefully assess the risk of suicidal behaviour. Drivers with psychotic disorders should undergo a careful fitness-to-drive assessment, irrespective of whether they are receiving antipsychotic medication.

## Supplementary Material

ckaf263_Supplementary_Data

## Data Availability

Research data are not shared. Sensitivity of the data and permissions from authorities do not allow that data is distributed elsewhere. Key Points Antipsychotics were present in 30% of deceased drivers with psychotic disorders Suicidal intentions were involved in fatal traffic accidents despite treatment with antipsychotics A comprehensive fitness-to-drive assessment should be conducted regardless of whether drivers with psychotic disorders are taking antipsychotic medication
