# Correction to: The role of psychotropic medication, alcohol, illicit drugs, and suicidal intention in fatal motor vehicle accidents involving drivers with psychotic disorders

**DOI:** 10.1093/eurpub/ckag156

**Published:** 2026-08-14

This is a correction to: Jussi Koskinen, Helinä Hakko, Pirkko Riipinen, Niina Sihvola, Anu-Helmi Halt, The role of psychotropic medication, alcohol, illicit drugs, and suicidal intention in fatal motor vehicle accidents involving drivers with psychotic disorders, *European Journal of Public Health*, Volume 36, Issue 2, April 2026, ckaf263, https://doi.org/10.1093/eurpub/ckaf263

In the originally published version of this manuscript, the supplementary data folder contained three different versions of the file.

This error has been corrected so that only the correct file remains.

