# Supplementary material for: The role of psychotropic medication, alcohol, illicit drugs, and suicidal intention in fatal motor vehicle accidents involving drivers with psychotic disorders
Source: Eur J Public Health. 2026 Feb 16;36(2):ckaf263. doi: 10.1093/eurpub/ckaf263 (PMC13017886; doi:10.1093/eurpub/ckaf263)
Supplement: ckaf263_Supplementary_Data [file ckaf263_supplementary_data.zip › 31-Dec-2025_010409_ejph-2025-03-sr-0203-File002.docx]

**Supplementary Table S1. The medications identified in post-mortem toxicology analyses ^a^ according to ATC-classification in the total study sample of 282 drivers (including 94 cases with a psychotic disorder and 188 controls) killed in fatal motor vehicle accidents (FMVA), n (%).**

| **ANY MEDICATION, n=91 (32.3% of total sample)**  **MEDICATION FOR THE NERVOUS SYSTEM (ATC: N-CODES), n=81 (28.7%)** | | |
| --- | --- | --- |
| **N01: Anesthetics, 14 (5.0%)**  Ketamine, n=1 (0.4%)  Lidocaine, n=14 (5.0%)  **N02A: Analgesics, opioids, n=5 (1.8%)**  Buprenorphine, n=2 (0.7%)  Oxycodone, n=1 (0.4%)  Tramadol, n=3 (1.1%)  **N02B: Analgesics, other, n=3 (1.1%)**  Paracetamol, n=3 (1.1%)  **N03 Antiepileptics, n=11 (3.9%)**  Carbamazepine/ hydroxy-carbamazepine, n=5 (1.8%)  Clonazepam, n=2 (0.7%)  Lamotrigine, n=2 (0.7%)  Oxcarbazepine, n=1 (0.4%)  Phenobarbital, n=1 (0.4%)  Primidone, n=1 (0.4%)  Valproic acid, n=3 (1.1%)  **N04 Anti-Parkinson drugs, n=4 (1.4%)**  Biperiden, n=1 (0.4%)  Orphenadrine, n=3 (1.1%) | **N05A: Antipsychotics, n=28 (9.9%)**  Aripiprazole, n=1 (0.4%)  Chlorpromazine, n=3 (1.1%)  Chlorprothixene, n=3 (1.1%)  Clopenthixol, n=1 (0.4%)  Levomepromazine n=3 (1.1%)  Litium, n=1 (0.4%)  Olanzapine, n=9 (3.2%)  Quetiapine, n=5 (1.8%)  Risperidone, n=2 (0.7%)  Thioridazine, n=4 (1.4%)  Zuclopethixol, n=1 (0.4%)  **N05B and N05C: Anxiolytics, hypnotics and sedatives, n=38** (13.5%)  Alprazolam, n=5 (1.8%)  Chlordiazepoxide /Chlopoxide/ Demoxepam, n=3 (1.1%)  Clobazam, n=1 (0.4%)  Diazepam/ Desmethyldiazepam, n=18 (6.4%)  Hydroxyzine, n=1 (0.4%)  Lorazepam, n=2 (0.7%)  Meprobamate, n=1 (0.4%)  Midazolam, n=1 (0.4%)  Oxazepam, n=22 (7.8%)  Temazepam, n=19 (6.7%)  Zolpidem, n=1 (0.4%)  Zopiclone, n=4 (1.4%) | **N06A: Antidepressants, n=22 (7.8%)**  Amitriptyline, n=1 (0.4%)  Citalopram/Norcitalopram, n=9 (3.2%)  Clomipramine, n=1 (0.4%)  Doxepin, n=1 (0.4%)  Fluoxetine, n=2 (0.7%)  Mianserin/Normianserin, n=2 (0.7%)  Mirtazapine, n=5 (1.8%)  Sertraline, n=1 (0.4%)  Venlafaxine, n=2 (0.7%)  **N07: Other nervous system drugs, n=1 (0.4%)**  Dextromethorphan, n=1 (0.4%) |
| **OTHER MEDICATION (REST OF THE ATC-CODES, n=22 (7.8%)** | | |
| **A: Alimentary tract and metabolism,**  **n=2 (0.7%)**  Metformin, n=1 (0.4%)  Metoclopramide, n=1 (0.4%)  **B: Blood and blood forming organs, n=2 (0.7%)**  Dipyridamole, n=1 (0.4%)  Warfarin, n=1 (0.4%) | **C: Cardiovascular system, n=14 (5.0%)**  Bisoprolol, n=4 (1.4%)  Ephedrine, n=1 (0.4%)  Metoprolol, n=6 (2.1%)  Propranonol, n=2 (0.7%)  Quinidine, n=1 (0.4%)  **G: Genito urinary system and sex hormones, n=1 (0.4%)**  Sildenafil, n=1 (0.4%) | **J: Anti-infectives for systemic us,e n=1 (0.4%)**  Fluconazole, n=1 (0.4%)  **M: Musculo-skeletal system, n=4 (1.4%)**  Ibuprofen, n=2 (0.7%)  Quinine, n=2 (0.7%)  **P: Antiparasitic products, insecticides and repellents, n=1 (0.4%)**  Hydroxychloroquine ,n=1 (0.4%)  **R: Respiratory system, n=1 (0.4%)**  Fexofenadine, n=1 (0.4%) |
| ^a^ Based on the post-mortem toxicology documents or other investigation documents of FMVA accident folders (The Finnish Crash Data institute, https://www.lvk.fi/en/the-finnish-crash-data-institute-oti/oti/) | | |

**Supplementary Table S2. The association of being a user of psychotropics, alcohol or illicit drugs at the time of Fatal Motor Vehicle Accident (FMVA) to the likelihood of suicidal intentions behind FMVA, and to the likelihood of accident or suicide being the official cause of death among the total study sample of FMVA drivers, including 94 cases and 188 controls.**

| **Findings of the post-mortem toxicology analyses** ^a^ | **Official cause-of-death category** ^c^ | | | | **Suicidal intentions**  **behind FMVA,** defined by the road-accident investigation team  **(yes vs. no)** ^d^ | |
| --- | --- | --- | --- | --- | --- | --- |
|  | **Suicide vs. other death categories** | | **Accident vs. other death categories** | |  |  |
|  | **OR (95% CI)^e^** | **p-value** | **OR (95% CI)^e^** | **p-value** | **OR (95% CI)^e^** | **p-value** |
| **Psychotropic medication, yes** |  |  |  |  |  |  |
| Antipsychotics | 5.09 (2.12–12.22) | < 0.001 | 0.21 (0.09–0.46) | < 0.001 | 6.47 (2.81–14.91) | < 0.001 |
| Antidepressants | 2.99 (1.08–8.24) | 0.035 | 0.45 (0.19–1.11) | 0.083 | 2.90 (1.11–7.61) | 0.030 |
| Anxiolytics, hypnotics and sedatives | 1.39 (0.54–3.61) | 0.499 | 0.60 (0.29–1.25) | 0.171 | 1.92 (0.84–4.40) | 0.125 |
| Mood stabilizers | 2.80 (0.71–11.10) | 0.143 | 0.26 (0.08–0.89) | 0.032 | 3.40 (0.95–12.16) | 0.060 |
|  |  |  |  |  |  |  |
| **Driving Under Influence of Alcohol (DUIA), yes** | 0.60 (0.25–1.43) | 0.245 | 2.65 (1.31–5.36) | 0.007 | 0.85 (0.40–1.78) | 0.660 |
|  |  |  |  |  |  |  |
| **Illicit drugs, yes** | ne^f^ |  | 1.18 (0.24–5.83) | 0.836 | ne^f^ |  |
|  |  |  |  |  |  |  |
| **Opioids, yes** | ne^f^ |  | ne^f^ |  | ne^f^ |  |
|  |  |  |  |  |  |  |
| ^a^ Based on the post-mortem toxicology documents or other investigation documents of FMVA accident folders (The Finnish Crash Data institute, https://www.lvk.fi/en/the-finnish-crash-data-institute-oti/oti/)  ^b^ Diagnosed with a psychotic disorder during the 5 years prior to the fatal accident based on the Care Register of Health Care (https://thl.fi/en/statistics-and-data/data-and-services/register-descriptions/care-register-for-health-care)  ^c^ Official death categories provided by Statistics Finland (https://stat.fi/meta/til/ksyyt_en.html)  ^d^ Based on the interviews and other background material of OTI data (The Finnish Crash Data institute, https://www.lvk.fi/en/the-finnish-crash-data-institute-oti/oti/)  ^e^ Unadjusted odds ratio (OR) with 95% confidence interval (CI).  ^f^ not estimable, the number of cases in these categories was too small for statistical analysis. | | | | | | |
